# Generation of tetracycline-controllable CYP3A4-expressing Caco-2 cells by the piggyBac transposon system

**DOI:** 10.1038/s41598-021-91160-z

**Published:** 2021-06-03

**Authors:** Moe Ichikawa, Hiroki Akamine, Michika Murata, Sumito Ito, Kazuo Takayama, Hiroyuki Mizuguchi

**Affiliations:** 1 Laboratory of Biochemistry and Molecular Biology, Graduate School of Pharmaceutical Sciences, Osaka University, 1-6 Yamadaoka, Suita, Osaka 565-0871 Japan; 2 GenoMembrane Co., Ltd., 2-3-18 Namamugi, Tsurumi-ku, Yokohama, Kanagawa 230-0052 Japan; 3 Laboratory of Hepatocyte Regulation, National Institutes of Biomedical Innovation, Health and Nutrition, Ibaraki, Osaka 567-0085 Japan; 4 Global Center for Medical Engineering and Informatics, Osaka University, Suita, Osaka 565-0871 Japan; 5 Integrated Frontier Research for Medical Science Division, Institute for Open and Transdisciplinary Research Initiatives (OTRI), Osaka University, Suita, Osaka 565-0871 Japan

**Keywords:** Biotechnology, Drug discovery

## Abstract

Caco-2 cells are widely used as an in vitro intestinal epithelial cell model because they can form a monolayer and predict drug absorption with high accuracy. However, Caco-2 cells hardly express cytochrome P450 (CYP), a drug-metabolizing enzyme. It is known that CYP3A4 is the dominant drug-metabolizing enzyme in human small intestine. In this study, we generated CYP3A4-expressing Caco-2 (CYP3A4-Caco-2) cells and attempted to establish a model that can simultaneously evaluate drug absorption and metabolism. CYP3A4-Caco-2 cells were generated by piggyBac transposon vectors. A tetracycline-controllable CYP3A4 expression cassette (tet-on system) was stably transduced into Caco-2 cells, thus regulating the levels of CYP3A4 expression depending on the doxycycline concentration. The CYP3A4 expression levels in CYP3A4-Caco-2 cells cultured in the presence of doxycycline were similar to or higher than those of adult small intestine. The CYP3A4-Caco-2 cells had enough ability to metabolize midazolam, a substrate of CYP3A4. CYP3A4 overexpression had no negative effects on cell proliferation, barrier function, and P-glycoprotein activity in Caco-2 cells. Thus, we succeeded in establishing Caco-2 cells with CYP3A4 metabolizing activity comparable to in vivo human intestinal tissue. This cell line would be useful in pharmaceutical studies as a model that can simultaneously evaluate drug absorption and metabolism.

## Introduction

Orally administered drugs are absorbed and metabolized in the small intestine and transferred to systemic circulating blood. Because various drug-metabolizing enzymes and drug transporters are highly expressed in the small intestine, pharmacokinetics in the small intestine has a great influence on the bioavailability of orally administered drugs^1^. In order to realize the efficient development of safe and effective orally ministered drugs, it is important to predict their intestinal absorption rate and intestinal first-pass effects in vitro. For this purpose, it is essential to establish a model that can simultaneously predict both drug absorption and metabolism.

There are several methods to evaluate the oral absorption and/or metabolism of drugs in the small intestine, but each has drawbacks^2–4^. Animal models such as those using Ussing Chambers and rodent intestinal tissue have the disadvantage that drug metabolizing enzymes and transporters differ between rodents and humans^2^. Artificial lipid membranes cannot predict the metabolism of oral drugs and their transport through the transporters^3^. Primary human small intestinal epithelial cells are difficult to obtain and culture^4^. Therefore, it is expected to develop an in vitro cell model that can accurately evaluate the absorption and metabolism of the drugs in the small intestine.

Caco-2 cells, a human colorectal adenocarcinoma cell line, have a epithelial-like morphology with a microvilli structure similar to that of the human small intestine, and form a tight junction by culturing on a cell culture insert^5,6^. Caco-2 cells express various drug transporters expressed in the human small intestine, thus they are widely used as an in vitro assay system for predicting gastrointestinal absorption (*Fa*) of orally administered drugs^7,8^. However, Caco-2 cells rarely express the drug-metabolizing enzyme, cytochrome P450 (CYP)^9^. Since CYP3A4 is a dominant drug-metabolizing enzyme in the human small intestine, accounting for approximately 80% of total intestinal CYP content^10^, development of a Caco-2 cell model that can predict CYP3A4-mediated drug metabolism in the small intestine is desirable. Many groups have tried to establish Caco-2 cells stably expressing CYP3A4, but only a few were reported, which used viral vectors, episomal vectors, and artificial chromosomes^11–15^. Brimer-Cline and Schuetz used adenoviral vectors to induce CYP3A4 expression in Caco-2 and LLC-PK1 cells^11^. Compared to the levels in LLC-PK1 cells, CYP3A4 expression levels in Caco-2 cells were low. In other reports, introduction of the CYP3A4 gene into Caco-2 cells using episomal vectors not only markedly affected cell growth, but resulted in unstable expression of CYP3A4^12,13^. Other groups developed Caco-2 cells co-expressing cytochrome P450 (CYPs) and NADPH-cytochrome P450 reductase (CPR) by using human artificial chromosome (HAC) vectors^14,15^. However, the metabolic activity for CYP3A4-substrate, midazolam, in these cells was equal to or slightly lower than that in human intestinal mucosa^14^. None of these approaches for establishing CYP3A4-expressing Caco-2 cells and/or the established cell line itself are widely used now.

In this study, we focused on "piggyBac transposon" isolated from *Trichoplusia ni* as a tool for overexpressing human CYP3A4. In the piggyBac transposon system, transposases cleave DNA regions between transposon-specific inverted terminal repeats (ITRs) sequences and insert them into TTAA chromosomal sites. By transfecting cells with a plasmid DNA carrying the piggyBac transposon system, the target gene can be integrated into the chromosome and be stably overexpressed^16,17^. The expression level of CYP3A4 varies among individuals^18^. We considered that a system which could control the degree of CYP3A4 expression would make it possible to account for individual differences in metabolism. Therefore, we attempted to establish a Caco-2 cell line that stably expresses CYP3A4 under the control of a tetracycline-controllable promoter using the piggyBac transposon system. We then evaluated whether the CYP3A4-expressing Caco-2 cells can be applied to pharmacokinetic studies.

## Results

### Generation of CYP3A4-expressing Caco-2 cells

**Figure 1:**
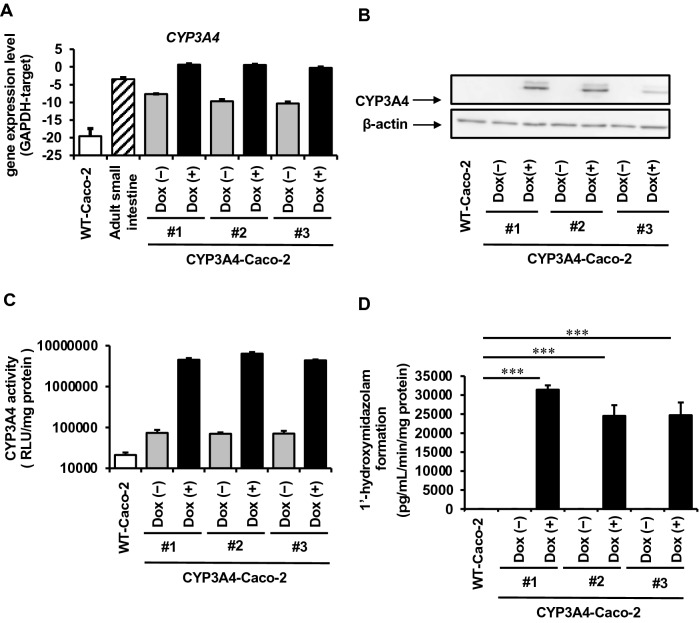
Generation of CYP3A4-expressing Caco-2 cells. The cells were seeded into the 12-well dishes. (**A**) The gene expression levels of CYP3A4 in WT-Caco-2 cells, human adult small intestine (adult small intestine), and CYP3A4-expressing Caco-2 cells (#1, #2, and #3) with or without Dox treatment were measured by real-time RT-PCR. (**B**) The protein expression levels of CYP3A4 in WT-Caco-2 cells and CYP3A4-expressing Caco-2 cells (#1, #2, and #3) with or without Dox treatment were measured by western blotting analysis. (**C**) The CYP3A4 activities in WT-Caco-2 cells and CYP3A4-expressing Caco-2 cells (#1, #2, and #3) with or without Dox treatment were examined using P450-Glo assay kit. (**D**) The CYP3A4-mediated drug metabolizing capacities in WT-Caco-2 cells and CYP3A4-expressing Caco-2 cells (#1, #2, and #3) with or without Dox treatment were evaluated by quantifying the metabolites of the CYP3A4 substrate, MDZ. The quantity of metabolites, 1′-OH MDZ, was measured by UPLC-MS/MS. The results are represented as means ± SD (*n* = 3, technical replicate). Statistical significances were evaluated by one-way ANOVA followed by Dunnett’s post-hoc test (***p < 0.001: compared with ‘‘WT-Caco-2’’). All data are represented as means ± S.D. (*n* = 3, technical replicate).

**Figure 2:**
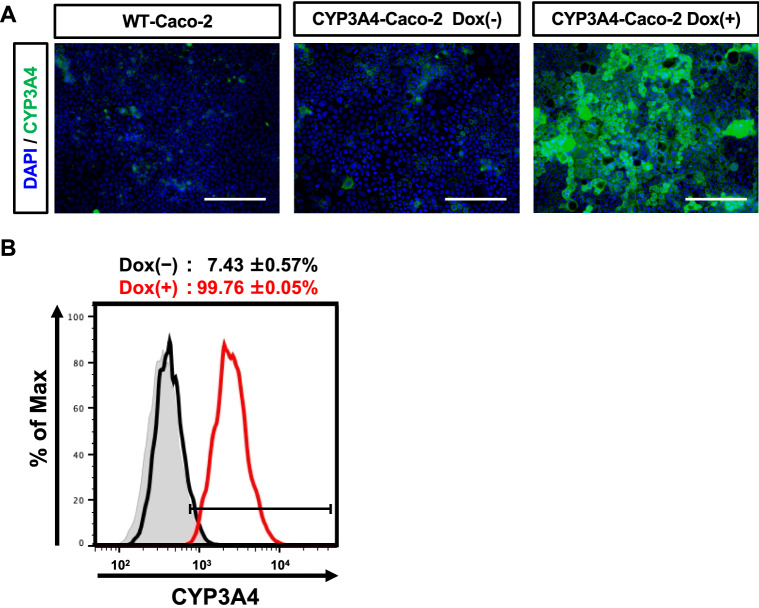
CYP3A4 expression analysis in CYP3A4-Caco-2 cells. The cells were seeded into the 12-well dishes. (**A**) Immunostaining analysis of CYP3A4 (green) was performed in the WT-Caco-2 cells and CYP3A4-expressing Caco-2 cells (#1) with or without Dox treatment (Dox (+) and Dox (−)). Nuclei were stained with DAPI (blue). Scale bar represents 200 μm. (**B**) The percentage of CYP3A4-positive cells in CYP3A4-Caco-2 cells (#1) with or without Dox treatment (Dox (+) and Dox (−)) was examined by FACS analysis.

It has been known that stable transfectants derived from Caco-2 cells cannot be obtained even by using virus vectors such as retrovirus, lentivirus, etc., as well as by a conventional plasmid transfection and drug selection. This is the reason why, though quite simple, a few studies about CYP3A4-expressing Caco-2 cells have been published^11–15,18^. To overcome these difficulties, we used piggyBac transposon system to forcibly integrate and express human CYP3A4 gene under the control of a tetracycline-controllable promoter into Caco-2 cells. By using piggyBac transposon system and blasticidin selection, we could obtain CYP3A4-expressing Caco-2 cells. The gene expression was analyzed in 3 cell lines (#1, #2, and #3). The gene expression levels of CYP3A4 in all Dox-treated Caco-2 cell lines were higher than those in human small intestine (Fig. 1A). Dox-treatment increased CYP3A4 protein expression and CYP3A4 activity in all Dox-treated Caco-2 cell lines (Figs. 1B, C and S1). The CYP3A4 activity in CYP3A4-Caco-2 cells was also examined using Midazolam (MDZ), which is a typical substrate of CYP3A4. Although CYP3A4 activity was below the detection limit in non-transfected wild type (WT)-Caco-2 cells and CYP3A4-Caco-2 cells without Dox-treatment, CYP3A4 activity could be detected in Dox-treated CYP3A4-Caco-2 cells (Fig. 1D). Immunostaining and flow cytometry analysis showed that percentage of CYP3A4-positive cells was increased by Dox-treatment (Fig. 2). We also confirmed that CYP3A4 activity was increased depending on the concentration of Dox (Fig. S2). Thus, we have succeeded in establishing Caco-2 cells expressing CYP3A4. In the following experiments, detailed examinations were performed using clone #1, which showed the highest CYP3A4 gene expression level.

### Temporal analysis of CYP3A4-expressing Caco-2 cells

**Figure 3:**
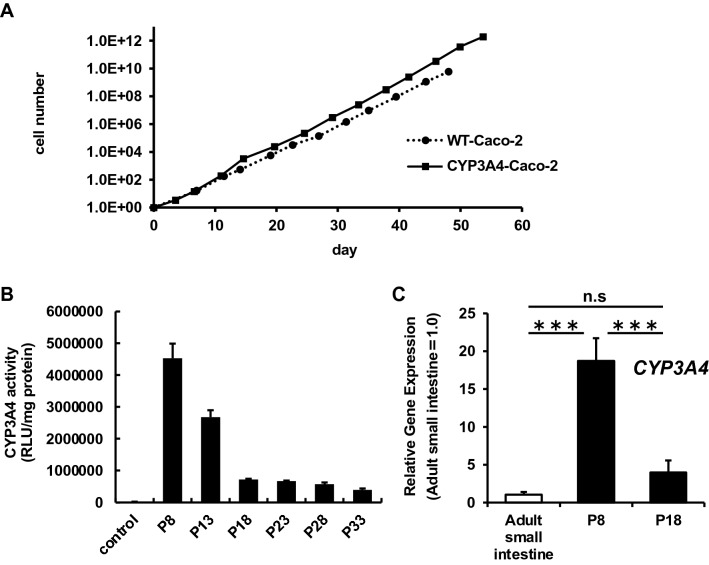
Temporal characterization of CYP3A4-Caco-2 cells. The cells were seeded into the 12-well dishes. (**A**) WT-Caco-2 cells and CYP3A4-Caco-2 cells (#1) were cultured and cell growth was analyzed by obtaining a cell count at each passage. The results are represented as means ± SD (*n* = 3, technical replicate). (**B**) Temporal CYP3A4 activity in WT-Caco-2 cells and Dox-treated CYP3A4-Caco-2 cells (passage number 8, 13, 18, 23, and 28) were examined using P450-Glo assay kit. (**C**) The gene expression levels of CYP3A4 in human adult small intestine (adult small intestine) and CYP3A4-Caco-2 cells (#1) (passage number 8 and 18) with Dox treatment were measured by real-time RT-PCR. Statistical significances were evaluated by one-way ANOVA followed by Tukey’s post-hoc tests (***p < 0.001). All data are represented as means ± S.D. (*n* = 3, technical replicate).

We investigated functional changes in CYP3A4-Caco-2 cells during several passages. The growth rate of CYP3A4-Caco-2 cells was similar to that of non-transfected WT-Caco-2 cells (Fig. 3A). The CYP3A4 activity in Dox-treated CYP3A4-Caco-2 cells was measured every 5th passages after the establishment (Fig. 3B). The CYP3A4 activity in CYP3A4-Caco-2 cells was gradually decreased, but the activity was stabilized after the 18th passage. The CYP3A4 gene expression levels were examined in CYP3A4-Caco-2 cells that had been passaged 18 times. The CYP3A4 gene expression levels in CYP3A4-Caco-2 cells even passaged 18 times were similar to those in human small intestine (Fig. 3C). These results suggested that the cell growth ability of CYP3A4-Caco-2 cells was normal and that CYP3A4 gene expression levels in CYP3A4-Caco-2 cells was similar to those of human small intestine. However, overexpression of CYP3A4 changed the gene expression of some other drug-metabolizing enzymes and transporters, probably due to random integration of multiple CYP3A4 expression cassettes into various regions of chromosomes in Caco-2 cells (Fig. S3).

### CYP3A4-expressing Caco-2 cells can evaluate both drug absorption and metabolism

**Figure 4:**
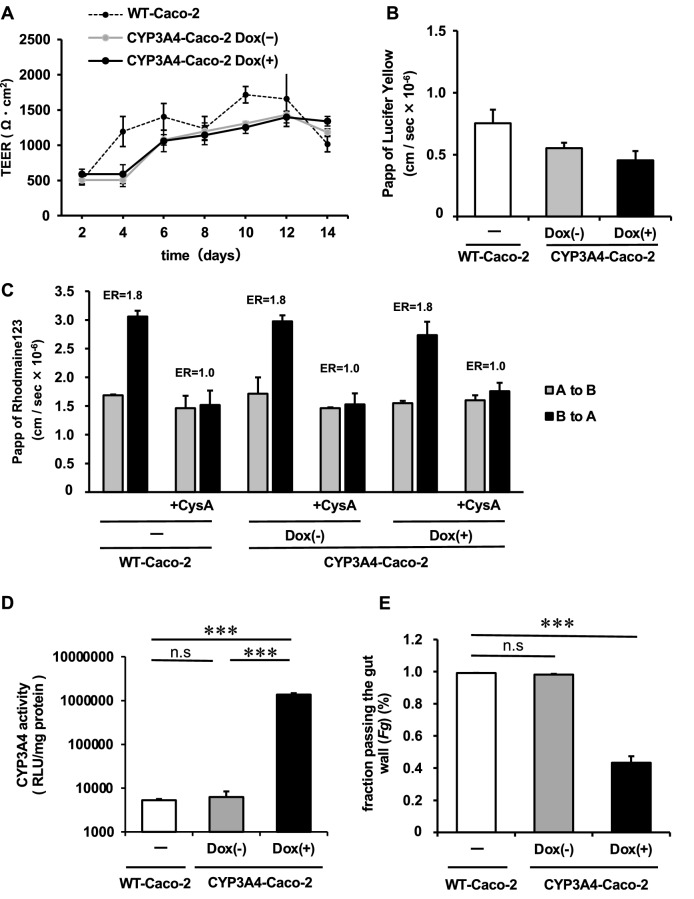
Barrier function and drug absorption capacity of CYP3A4-expressing Caco-2 cells monolayer. The cells were seeded into the cell culture inserts. (**A**) Transepithelial electrical resistance (TEER) values of the WT-Caco-2 cells and CYP3A4- Caco-2 cells with or without Dox treatment (Dox (+) and Dox (−)) were measured by Millicell-ERS2. (**B**) The apical-to-basolateral permeability of Lucifer Yellow across the WT-Caco-2 cells and CYP3A4-Caco-2 cells with or without Dox treatment (Dox (+) and Dox (−)) was measured. (**C**) The apical-to-basolateral permeability of rhodamine123 across the WT-Caco-2 cells and CYP3A4-Caco-2 cells with or without Dox treatment (Dox (+) and Dox (−)) in the presence or absence of 10 μM cyclosporine A (CysA; an P-gp inhibitor) was measured. Efflux ratio (ER) was used to evaluate the function of P-gp. The calculation was performed using the following equation. ER = *Papp* (B to A)/*Papp* (A to B) (**D**) The CYP3A4 activities of the WT-Caco-2 cells and CYP3A4-Caco-2 cells with or without Dox treatment (Dox (+) and Dox (−)) were examined using P450-Glo assay kit. Dox-treatment was performed for 7 days after the monolayer formation. Statistical significances were evaluated by one-way ANOVA followed by Tukey’s post-hoc tests (***p < 0.001). (**E**) The in vitro *Fg* of MDZ in the WT-Caco-2 cells and CYP3A4-Caco-2 cells with or without Dox treatment (Dox (+) and Dox (−)) was measured. CYP3A4-mediated drug metabolizing capacities were evaluated by quantifying MDZ and 1′-OH MDZ in the apical chamber, basolateral chamber, and cells. The *Fg* was calculated as described in the Materials and Methods section. Statistical significances were evaluated by one-way ANOVA followed by Dunnett’s post-hoc test (***p < 0.001: compared with ‘‘WT-Caco-2’’). The results are represented as means ± SD (*n* = 3, technical replicate).

To investigate whether the established CYP3A4-Caco-2 cells can be applied to pharmacokinetic research, the drug metabolism and transport abilities were examined after culturing CYP3A4-Caco-2 cells on cell culture insert for 14 days and Dox-treatment for 7 days. To evaluate the barrier function of CYP3A4-Caco-2 cell monolayer, transepithelial electrical resistance (TEER) was measured and the membrane permeability coefficient (Papp) of Lucifer Yellow, a paracellular permeability marker, was calculated. At day 6 after the cell seeding, the TEER value of CYP3A4-Caco-2 cells reached 1000 Ω·cm^2^ (Fig. 4A). Papp of Lucifer Yellow was 1.0 cm/s × 10^−6^ or less in both non-transfected WT-Caco-2 cells and CYP3A4-Caco-2 cells (Fig. 4B). CYP3A4 transfection reduced TEER, but TEER values in CYP3A4-Caco-2 cells were well above 100 Ω·cm^2^, which was sufficient to indicate that the monolayers had formed. Importantly, there was no difference in TEER values between CYP3A4-Caco-2 cells with and without Dox-treatment. Therefore, it was indicated that overexpression of CYP3A4 did not negatively affect the barrier function of Caco-2 cells. In addition, to examine whether CYP3A4-Caco-2 cells have transporter activity, we performed a P-gp transport assay in a CYP3A4-Caco-2 monolayer by using rhodamine123 (P-gp substrate). P-gp is an excretory transporter, and it is known to play an important role in the intestinal absorption and excretion of various drugs, such as digoxin and verapamil^19,20^. The basolateral-to-apical (B to A) Papp value of rhodamine123 was decreased by cyclosporine A (an P-gp inhibitor), and P-gp activity was confirmed in both WT-Caco-2 cells and CYP3A4-Caco-2 cells with or without Dox (Fig. 4C). CYP3A4 activity was significantly increased even when Dox-treatment was performed after the monolayer formation (Fig. 4D). Furthermore, to examine whether the human in vivo* Fg* can be predicted by CYP3A4-Caco-2 cells, the CYP3A4 substrate passing CYP3A4-Caco-2 cells were evaluated by quantifying MDZ and 1-OH MDZ in apical chamber, basolateral chamber, and the cells after 60-min incubation (Fig. 4E). The *Fg* value (*Fg* = 0.43) of MDZ in Dox-treated CYP3A4-Caco-2 cells was lower than that in human intestinal tissue (*Fg* = 0.57–0.62)^21,22^, while that in non-transfected WT-Caco-2 cells and CYP3A4-Caco-2 cells without Dox treatment was 0.99. This result suggested that Dox-treated CYP3A4-Caco-2 cells metabolized more MDZ than in vivo human intestinal tissue. Together, these results indicate that the established CYP3A4-Caco-2 cells are a model that can evaluate both drug absorption and metabolism.

## Discussion

In this study, we succeeded in establishing Caco-2 cell lines stably expressing CYP3A4 by using piggyBac transposon system. The CYP3A4-expressing Caco-2 cells established in the previous reports had insufficient CYP3A4 activity and CYP3A4 expression did not last for a long period, probably due to the silencing of CYP3A4 gene^13^. It is not clear why the stable expression of CYP3A4 was possible by using the PiggyBac transposon system. We assume that certain amounts of foreign (CYP3A4) genes are protected from the silencing. Inclusion of insulator sequence might have positive effect on high and stable CYP3A4 expression. Since the CYP3A4 activity in CYP3A4-Caco-2 cells was decreased from passage 8 to passage 18 (Fig. 3B), gene silencing would occur in this period. Importantly, after passage 18, CYP3A4 activity was stable and CYP3A4 gene expression level was comparable to that of adult small intestine, which means its level was high enough. The *Fg* value (*Fg* = 0.43) of MDZ in Dox-treated CYP3A4-Caco-2 cells (passage 18) was lower than that in human intestinal tissue (*Fg* = 0.57–0.62)^21,22^ (Fig. 4E), suggesting that CYP3A4-Caco-2 cells (passage 18) had sufficient drug metabolizing activity. It is assumed that by using piggyBac transposon, more exogenous genes were inserted into the various regions of the host chromosomes as compared with other gene transfer methods, and thus the Caco-2 cells with high CYP3A4 activity could be established. To the best of our knowledge, CYP3A4-Caco-2 cells in the present study showed the highest CYP3A4 activity among the previously published studies^11–15^. Takenaka et al. co-expressed NADHP-cytochrome P450 reductase as well as CYP3A4 in Caco-2 cells using a human artificial chromosome^14^. Our study clearly showed that exogenous expression of NADPH-cytochrome P450 reductase is not required for the establishment of Caco-2 cells with adequate CYP3A4 metabolizing activity.

In this study, the Tet-On system was used to control CYP3A4 expression, and the CYP3A4 expression level could be manipulated in a Dox concentration-dependent manner (Fig. S2). Therefore, it might be possible to establish an in vitro evaluation system that can reflect individual differences in CYP3A4 expression level in the human small intestine by adjusting the Dox concentration. Among the 10 major genes involved in pharmacokinetics other than CYP3A4, the gene expression levels were changed in 5 genes in CYP3A4-Caco-2 cells (Fig. S3). This might be due to the random insertion of CYP3A4 genes by the piggyBac transposon system. Recently, the novel piggyBac transposon system has been reported that can target the insertion site by fusing transposase with proteins such as zinc finger nuclease (ZFP), transcription activator-like effector nuclease (TALEN), and catalytically dead SpCas9-HF1 (dCas9)^23–25^. By using these systems, the Caco-2 cell line with less off-target effect may be established.

In recent years, there have been a series of reports describing the establishment of intestinal models using human iPS cells and organoids^26–28^. However, the establishment and maintenance of human iPS cell-derived intestinal epithelial cells and human intestinal organoids require specialized techniques and are expensive and often hindered by ethical restrictions. Therefore, they have not yet been put to practical use in drug discovery. On the other hand, Caco-2 cells are relatively easy to handle, and have no ethical restrictions. In addition, since Caco-2 cells have been used for many years in in vitro oral absorption evaluation systems, if a new Caco-2 cell model could be constructed, it would be easily introduced into pharmacokinetic studies.

In summary, we established CYP3A4-expressing Caco-2 cells with enough drug metabolizing activity. This easy-to-use cell line would be a useful tool for the evaluation of in vitro oral absorption and metabolism.

## Materials and methods

### Caco-2 cell culture and differentiation

The human colorectal adenocarcinoma cell line, Caco-2 (HTB-37, ATCC), was obtained from the American Type Culture Collection. Caco-2 cells were cultured with minimum essential medium (Sigma-Aldrich) containing 10% fetal bovine serum (FBS), 1 × MEM Non-Essential Amino Acid Solution (NEAA), 100 μg/mL streptomycin, and 100 U/mL penicillin, and 1 × GlutaMAX (Thermo Fisher Scientific). Caco-2 cells were cultured for 14 days after they reached confluence for the differentiation.

### Plasmid

The piggyBac transposon vector system from System Biosciences (PB533A-2) was used in this study. First, the expression cassette containing TRE3G-CMV promoter, blasticidin resistant gene, and EF1α promoter-driven Tet-On-3G gene, which was generated by using a standard GENE SYNTHESIS service (GENEWIZ), was inserted into PB533A-2 using In-Fusion HD cloning kit (Takara Bio), resulting in pPB-TRE3G. The sequences of TRE3G and Tet-on-3G genes were obtained from Tet-On 3G inducible expression system (Takara Bio). The human CYP3A4 gene was amplified by PCR using pAdHM4-E4-122aT^29^ in which the human CYP3A4 gene from human liver cDNA was cloned. Then, the human CYP3A4 gene was inserted into pPB-TRE3G using In-Fusion HD cloning kit, resulting in pPB-TRE3G-CYP3A4 (Fig. S4).

### Generation of CYP3A4-expressing Caco-2 cells

pPB-TRE3G-CYP3A4 and piggyBac transposase vectors (System Biosciences) were co-transfected into Caco-2 cells by Lipofectamine 2000 reagent (Thermo Fisher Scientific), and then drug selection was performed using Blasticidin (FUJIFILM Wako). Resulting colonies were cultured. For the induction of CYP3A4 expression, the cells were cultured for 1 μg/ml doxycycline (Dox) for 7 days. CYP3A4-expressing Caco-2 cells passaged for 10–18 times were used in the experiments, except in Fig. 3B.

### Real-time RT-PCR

Real-time RT-PCR was performed according to our previous report^30^. Briefly, total RNA was isolated from Caco-2 cells and their derivatives using ISOGENE (NIPPON GENE). cDNA was synthesized using 500 ng of total RNA with a Superscript VILO cDNA synthesis kit (Thermo Fisher Scientific). Total RNA of Human Adult Normal Tissue: Small Intestine was purchased from BioChain Institute. Real-time RT-PCR was performed with SYBR Green PCR Master Mix (Thermo Fisher Scientific) using a StepOnePlus real-time PCR system (Thermo Fisher Scientific). The relative quantitation of target mRNA levels was performed using the 2^−ΔΔCT^ method. The values were normalized by those of the housekeeping gene, *glyceraldehyde 3-phosphate dehydrogenase* (*GAPDH*). PCR primer sequences were obtained from PrimerBank (https://pga.mgh.harvard.edu/primerbank/).

### Immunocytochemistry

Immunocytochemistry was performed according to our previous report^30^. Briefly, Caco-2 cells and their derivatives were fixed with 4% paraformaldehyde (FUJIFILM Wako) in PBS for 10 min. After blocking the cells with PBS containing 2% bovine serum albumin (BSA) and 0.2% Triton X-100 for 20 min, the cells were incubated with primary antibodies at 4 °C overnight, and finally, with secondary antibodies at room temperature for 1 h. All antibodies used in this report are described in Supplemental Table 1.

### Western blotting

Western blotting was performed according to our previous report^31^. Briefly, the cells were homogenized with RIPA Lysis and Extraction buffer (Thermo Fisher Scientific) containing a protease inhibitor mixture (Sigma Aldrich). After being frozen and thawed, the homogenates were centrifuged at 15,000 *g* at 4 °C for 10 min, and the supernatants were collected. The lysates were subjected to SDS-PAGE on 7.5% polyacrylamide gel, and then transferred onto polyvinylidene fluoride membranes (Millipore). The reaction was blocked with 1% skim milk in Tris Buffered Saline (TBS) containing 0.1% Tween 20 at room temperature for 1 h. The membranes were incubated with primary antibodies at 4 °C overnight, followed by reaction with secondary antibodies at room temperature for 1 h. The band was visualized by Chemi-Lumi One Super (Nakalai Tesque) and the signals were read using a LAS-4000 imaging system (FUJIFILM). All antibodies used in this report are described in Supplemental Table 1.

### CYP3A4 activity

To measure the CYP3A4 activity, the Lytic assay was performed by using a P450-Glo™ CYP3A4 Assay with Luciferin-IPA (Promega) according to the manufacturer’s instructions. The luminescence was read by a Lumat LB 9507 (Berthold Technologies). The CYP3A4 activity was normalized with the protein content per well, which was evaluated with the Pierce BCA Protein Assay Kit (Thermo Fisher Scientific).

UPLC-MS/MS analysis was also performed to examine the CYP3A4 activity according to our previous report^30^. Briefly, Caco-2 cells and their derivatives were cultured with medium containing 5 μM midazolam (MDZ, FUJIFILM Wako). After the treatment with MDZ, the supernatant was collected at 30 min, and then immediately mixed with two volumes of acetonitrile (FUJIFILM Wako). The supernatant was analyzed by UPLC-MS/MS to measure the concentrations of the metabolite, 1′-hydroxymidazolam (1′-OH MDZ), according to each standard followed by normalization to the protein content per well.

### Flow cytometry

Caco-2 cells and their derivatives were fixed with 4% paraformaldehyde at 4 °C for 10 min, and then incubated with primary antibodies, followed by secondary antibodies. MACSQuant Analyzer (Miltenyi Biotec) and FlowJo software (FlowJo LLC, http://www.flowjo.com/) were used for analysis. All antibodies used in this report are described in Supplemental Table 1.

### Transepithelial electrical resistance (TEER) measurements

TEER values were measured by Millicell-ERS2 (Merck Millipore). Caco-2 cells and their derivatives were cultured on BD Falcon cell culture inserts (6 well plate, 0.4 μm pore size, 2.0 × 10^6^ pores/cm^2^, BD Biosciences) from day 0 of differentiation. The raw data were converted to Ω × cm^2^ based on the culture insert area (4.2 cm^2^).

### Calculation of apparent permeability

The apparent permeability coefficient (*Papp*) in transport assay was calculated according to the following equation.

$${\text{Papp}} =\updelta {\text{Cr}}/\updelta {\text{t}} \times {\text{Vr}}/\left( {{\text{A}} \times {\text{C}}_{0} } \right)$$

δCr/δt = permeability rate (δCr = final receiver concentration, δt = assay time); Vr = receiver volume; A = transwell growth area; C_0_ = initial concentration in the donor compartment.

### Lucifer Yellow permeability tests

Caco-2 cells and their derivatives, which were cultured on the cell culture inserts, were rinsed with HBSS. The 1.5 mL of HBSS containing 100 μM Lucifer Yellow CH Dipotassium Salt (FUJIFILM Wako) was added to the apical chamber, and 3.0 mL of HBSS was also added to the basolateral chamber. After 90 min incubation at 37 °C, the solution was collected from the basolateral chamber. The Lucifer Yellow fluorescent signal was measured with a fluorescence plate reader (TriStar LB 941, Berthold Technologies) using 428 nm excitation and 535 nm emission filters. Lucifer Yellow concentrations were calculated using the standard curve generated by serial dilution of Lucifer Yellow.

### Rhodamine123 permeability tests

Caco-2 cells and their derivatives, which were cultured on the cell culture inserts, were rinsed with HBSS. The cells were preincubated for 30 min with HBSS in the presence or absence of 10 µM Cyclosporin A (Sigma-Aldrich) as the inhibitor. To measure apical to basolateral (A to B) permeability, the 1.5 mL of HBSS containing 10 μM Rhodamine123 (Sigma-Aldrich) was added to the apical chamber, and 3.0 mL of HBSS was also added to the basolateral chamber. To measure basolateral to apical (B to A) permeability, the 3.0 mL of HBSS containing 10 μM Rhodamine123 (Sigma-Aldrich) was added to the basolateral chamber, and 1.5 mL of HBSS was also added to the apical chamber. After 90 min incubation at 37 °C in the presence or absence of the inhibitor, the solution was collected from the basolateral (A to B) or apical (B to A) chamber. The Rhodamine123 fluorescent signal was measured with a fluorescence plate reader (TriStar LB 941, Berthold Technologies) using 428 nm excitation and 535 nm emission filters. Rhodamine123 concentrations were calculated using the standard curve generated by serial dilution of Rhodamine123.

The ER of rhodamine 123 was calculated according following equation.

$${\text{ER}} = Papp\left( {{\text{B}}\;{\text{to}}\;{\text{A}}} \right)/Papp\left( {{\text{A}}\;{\text{to}}\;{\text{B}}} \right)$$

### Determination of *Fg* value *of MDZ*

*Fg* value of MDZ was determined according to our previous report^30^. Briefly, Caco-2 cells and their derivatives were cultured on the cell culture inserts. The 1.5 mL of HBSS containing 3 μM MDZ was added to the apical chamber, and 2.6 mL of HBSS containing 0.1% DMSO was added to the basolateral chamber. After incubation at 37 °C for 60 min, the solution was collected from the basolateral or apical chamber. The cells were also collected at the same time, and the collected cell solution was disrupted with BIORUPTOR. The amounts of MDZ and 1′-OH MDZ in each fraction were determined using UPLC-MS/MS as described above. The amounts of 1′-OH MDZ found in the apical, basolateral, and cell compartments were summed and entered into the following equation along with the amount of MDZ found in the basolateral and cell compartments to calculate the *Fg* value.

$$Fg\left( \% \right) = {\text{MDZ}}\left( {{\text{basolateral}} + {\text{cell}}} \right)/\left[ {{\text{MDZ}}\left( {{\text{basolateral}} + {\text{cell}}} \right) + \sum {\left( {1^{\prime } - {\text{OH}}\;{\text{MDZ}}} \right)} } \right]$$

It is known that *Fg* values of human in vivo are 0.57–0.62^21,22^.

## Supplementary Information

Supplementary Information 1.
