# Supplementary material for: Generation of tetracycline-controllable CYP3A4-expressing Caco-2 cells by the piggyBac transposon system
Source: Sci Rep. 2021 Jun 3;11:11670. doi: 10.1038/s41598-021-91160-z (PMC8175591; doi:10.1038/s41598-021-91160-z)
Supplement: Supplementary file 1 — Supplementary Information 1. [file 41598_2021_91160_MOESM1_ESM.pdf]

SUPPLEMENTAL INFORMATION

Title

Generation of tetracycline-controllable CYP3A4-expressing Caco-2 cells by the piggyBac transposon system

Authors

Moe Ichikawa, Hiroki Akamine, Michika Murata, Sumito Ito, Kazuo Takayama, Hiroyuki Mizuguchi

Supplemental Table

Supplemental Table 1

Supplemental Figures

Supplemental Figures S1-S4

Supplemental Table 1 The Antibodies used in this study

Antibody	Supplier	catalogue number
Anti-CYP3A4 antibody	Santa Cruz Biotechnology	sc-27639
Donkey anti-goat IgG Secondary Antibody, Alexa Fluor 488 conjugate	Thermo Fisher Scientific	A-11055
Anti-Goat IgG (whole molecule)–Peroxidase antibody produced in rabbit affinity isolated antibody, buffered aqueous solution	Sigma-Aldrich	A5420
Monoclonal Anti-β-Actin antibody produced in mouse clone AC-15, ascites fluid	Sigma-Aldrich	A5441
Anti-mouse IgG, HRP-linked Antibody	Cell signaling technology	7076S

Supplementary Figure 1

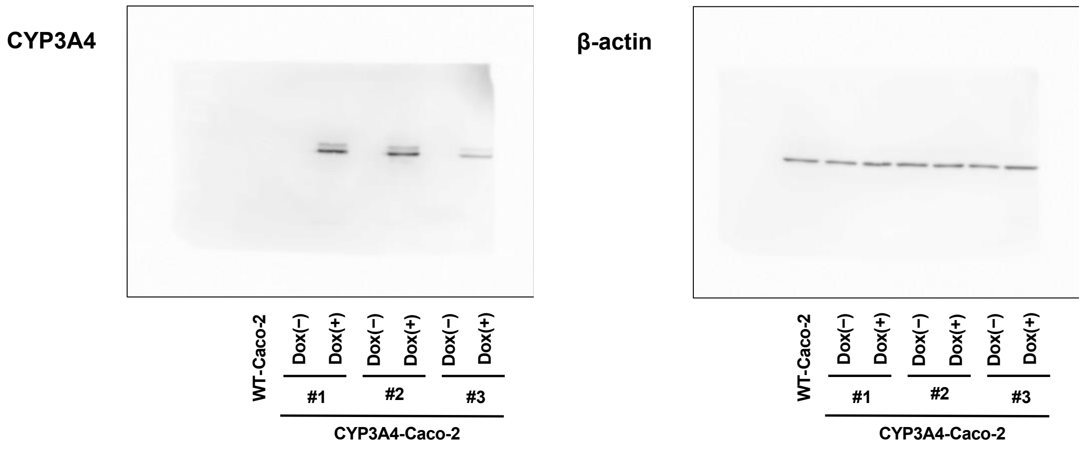

Figure S1 Original, unedited images for full length blots used to generate the figure 1B

These are images of the same membrane. After re-probing, I changed the antibodies and took pictures.

Supplementary Figure 2

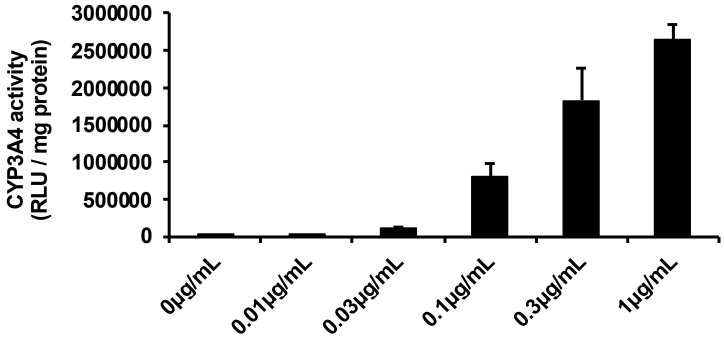

Figure S2 CYP3A4 activity can be regulated by controlling the Dox concentration

CYP3A4-expressing Caco-2 cells seeded into the 12-well dishes were cultured with various concentration of Dox for 7 days. The CYP3A4 activities in Dox-treated CYP3A4-expressing Caco-2 cells were examined using P450-Glo assay kit. The results are represented as means ± SD (n=3, technical replicate).

Supplementary Figure 3

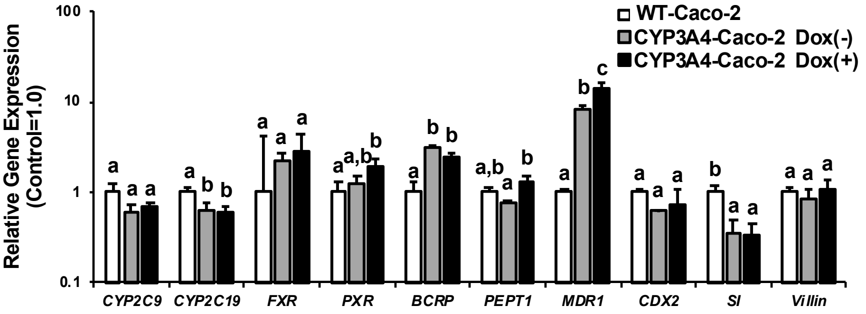

Figure S3 Gene expression analysis of intestinal markers in CYP3A4-Caco-2 cells

The gene expression levels of various intestinal markers in WT-Caco-2 cells and CYP3A4-Caco-2 cells, seeded into the 12-well dishes, with or without Dox treatment were measured by real-time RT-PCR. The gene expression levels of intestinal markers in WT-Caco-2 cells were taken as 1.0. Statistical significances were evaluated by one-way ANOVA followed by Tukey's post-hoc tests ($p < 0.05$). Groups that do not share the same letter are significantly different from each other. The results are represented as means ± SD (n=3, technical replicate).

Supplementary Figure 4

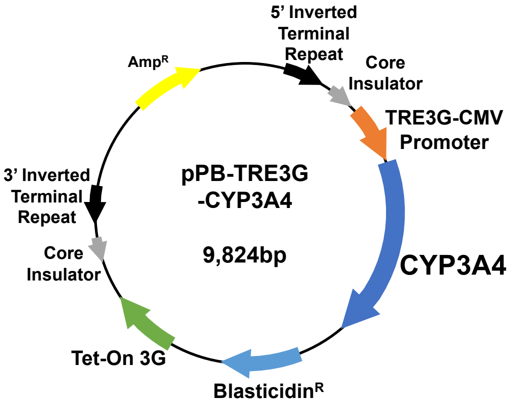

Figure S4 The vector map of pPB-TRE3G-CYP3A4

TRE3G-CMV promoter-driven CYP3A4 gene, blasticidin resistant gene-expression cassette, and EF1α promoter-driven Tet-On-3G gene are franked by insulator sequences and PiggyBac (PB) terminal repeats.
